# An Enhanced Cooling Method for Power Modules on All-Electric Ships Based on Parameter Optimization and Special-Shaped Design of Sintered Heat Pipes

**DOI:** 10.3390/mi16111197

**Published:** 2025-10-22

**Authors:** Binyu Wang, Ting Lu, Qisheng Wu, Bobin Yao, Hongwei Zhang, Xiwei Zhou, Weiyu Liu

**Affiliations:** 1 School of Electronics and Control Engineering, Chang’an University, Xi’an 710064, China; wangbinyu@chd.edu.cn (B.W.); qshwu@chd.edu.cn (Q.W.); b.b.yao@chd.edu.cn (B.Y.); engraved86@126.com (H.Z.); 2 School of Automation Science and Electrical Engineering, Beihang University, Beijing 100191, China; luting@buaa.edu.cn

**Keywords:** all-electric propulsion ships, multichip power module, hotspot chip temperature, heat flux, air-cooling heatsink, heat pipe, enhanced cooling, parameter optimization, special-shaped design

## Abstract

This paper proposes an enhanced cooling method for multi-chip power modules (e.g., in MMC inverters) with uneven power loss in all-electric propulsion ships based on sintered heat pipe parameter optimization and special-shaped design. First, five key parameters of straight sintered heat pipes were optimized: placement directly under hotspot chips, 10 mm in diameter, quantity matching the number of hotspot chips, length equal to the heatsink side length, and direction perpendicular to heatsink fins. Then, a C-shaped heat pipe was designed using the parallel thermal resistance principle, which forms two parallel low-thermal-resistance paths and outperforms conventional U-shaped ones. Finite element simulations showed that the hotspot temperature of the conventional heatsink was 91.26 °C, while it dropped to 87.35 °C with optimized straight heat pipes and further to 80.85 °C with C-shaped ones. Experiments verified an 11.65% temperature reduction (from 86.7 °C of conventional heatsinks to 76.6 °C of C-shaped heat pipe heatsinks). This method effectively lowers hotspot temperatures, reduces device failure rates, improves the thermal reliability of power modules, and provides a generalized design methodology for heatsinks of various power electronic converters.

## 1. Introduction

**Figure 1:**
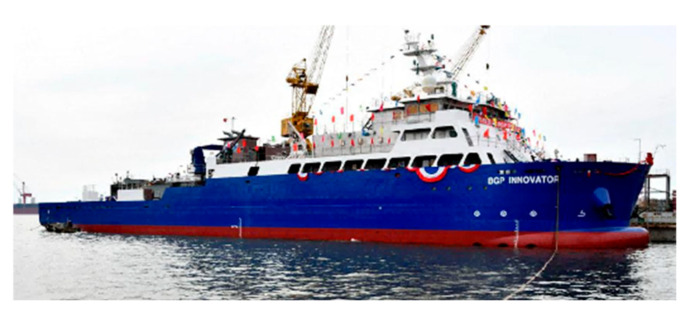
*BGP Innovator* [3].

Currently, with the advantages of high propulsion efficiency, low vibration and noise, high maneuverability, and cabin space savings, all-electric propulsion has become a natural requirement of modern ships [1,2]. Figure 1 [3] shows the *Bureau of Geophysical Prospecting (BGP) Innovator*, which adopted all-electric propulsion technology. This ship was produced by China and launched in 2022. Additionally, other countries are also actively developing high-performance all-electric propulsion ships.

Motor drive devices based on power electronic converters are the core components of an all-electric propulsion ship. The performance of power electronic converters determines the performance of all-electric propulsion ships. As long-term ocean voyages make maintenance difficult, thermal reliability on a long time scale is one of the most important parameters of power electronic converters on ships. Due to the special working principles of some converters, the power loss distribution in multichip power modules is unbalanced. This will cause higher heat fluxes and junction temperatures in some chips in the power modules. As is known, a higher junction temperature will reduce the lifespan of chips. The hotspot chips with the lowest lifespan will shorten the lifespan of the power modules [4]. Furthermore, current research results indicate that a 10 °C increase in junction temperatures will double a device’s failure rate. Hence, in order to reduce the hotspot chip’s temperature and meet reliability targets, heatsink optimization design has become increasingly important [5]. 

With advantages of high modularity, good scalability, high efficiency, and superior harmonic performance, modular multilevel converters (MMCs) are recognized as one of the most attractive converters. Furthermore, MMC-based motor drive devices are very promising when used on ships because they have no requirement for large phase-shifting transformers; moreover, they are easy to transport and install, have a common DC bus, and can be easily expanded to a back-to-back structure [6,7]. The voltage and power level of ship motors are much higher, and MMC is superior to two-level inverters in this field. 

Unfortunately, MMC-based motor drive devices always operate at high power factors, being the most typical example of devices with hotspot chips. The lower switches of the half-bridge submodules suffer from substantially higher power loss than other devices. The root cause of unbalanced power loss distributions is that the arm current exhibits a large DC bias when the power factor is high [8]. Compared with an MMC-based flexible DC transmission device, the power level of an MMC motor drive device is much lower. Hence, welded power modules with air-cooling devices are used in MMC motor drive devices like SINAMICS PERFECT HARMONY GH150 from Siemens [9] and the prototype created at Ohio State University [10]. Air-cooling devices have the advantages of low cost and easy implementation. However, an air-cooling heatsink has very limited cooling capacity since the thermal conductivity and heat transfer coefficient of air are much lower than those of most liquids. Hence, optimizing an air-cooling heatsink is necessary for hotspot chip temperature reduction. However, as far as we know, increasing the heatsink volume will reduce the efficiency of the heatsink, while increasing the air flow rate will increase the cost, energy consumption, and noise of the fans.

Researchers have made some efforts to optimize air-cooling devices. An automated air-cooling heatsink optimization method for power modules using a genetic algorithm is proposed in [5]. The cross-section of the heatsink is optimized, and the maximum junction temperature can be reduced by 6%. A comprehensive optimization method combining a heatsink and an inductance filter was proposed in [11].

By adopting the phase change heat transfer mode, heat pipes have substantially higher axial thermal conductivities. Moreover, heat pipes have the advantages of low cost, small size, easy processing, no energy consumption, and long-term operation. Hence, a heat pipe is an ideal element for optimizing an air-cooling heatsink. In recent years, many studies have been devoted to heat pipe heatsink research. A heat pipe heatsink design for next next-generation CPU modules is presented in [12]. The heat-spreading characteristics of a heatsink of the IGBT module with an embedded heat pipe versus a conventional heatsink are reported in [13,14]. The thermal performance and reliability of a heatsink with heat pipes designed for use with power electronic modules were evaluated by statistical methods in [15]. An electro-thermal model for an IGBT module with a heat pipe heatsink is proposed in [16]. A lumped RC thermal model is proposed in [17] in order to simulate the cooling of an IGBT module by heat pipe systems. Two different heat pipe embedding technologies are compared in [18].

**Figure 2:**
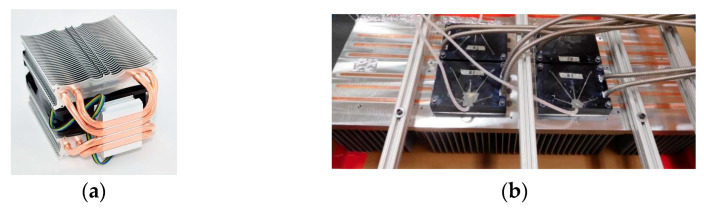
(**a**) Heat pipe heatsink for a computer CPU and (**b**) power module heatsink with straight heat pipes.

A common computer CPU heatsink is shown in Figure 2a. The structure of a U-shaped heat pipe is simple and sufficient for scientific use. The heatsink with embedded straight heat pipes for power modules mentioned in the existing literature is shown in Figure 2b [18]. It can be seen that the base of the heatsink is filled with as many heat pipes as possible, but this lacks a theoretical optimization process. And there are a few existing studies about optimization design theories for the heat pipes used in power module heatsinks.

Based on the above review of the literature, the objectives of this study are as follows:

1. To establish a theoretical framework and optimization procedure for the key physical parameters of sintered heat pipes;
2. To propose and theoretically justify a novel, specially shaped heat pipe design;
3. To formulate the findings into a convenient, generalized design methodology that can be applied to optimize heatsinks for various power electronic converters;
4. To verify the proposed enhanced cooling method through a comprehensive case study involving both finite element simulations and experiments.

In this paper, an enhanced cooling method of power modules on all-electric propulsion ships based on parameter optimization and the special-shaped design of sintered heat pipes is proposed. This can be used as a general design theory of air-cooling heatsinks for multichip power modules with hotspot chips. The rest of this paper is organized as follows. The parameter optimization and special-shaped design methodology of sintered heat pipes are researched in Section 2. The heatsink optimization case of the MMC inverter is conducted in Section 3. The experimental verification is shown in Section 4. Section 5 concludes the whole article.

## 2. Research on Enhanced Cooling Methods

### 2.1. Problems of Traditional Heatsinks

**Figure 3:**
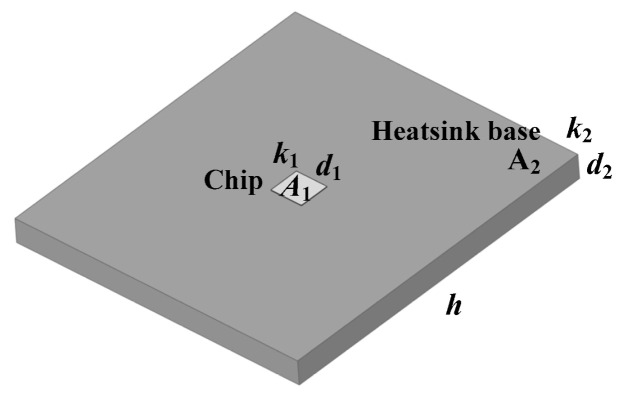
A chip located above a heatsink base.

The problems of traditional heatsinks can be explained from the perspective of spreading thermal resistance. A chip and a heatsink base are shown in Figure 3, and the chip is located above the base. The area, thermal conductivity, and thickness of the chip and base are *A*_1_, *k*_1_, and *d*_1_ and *A*_2_, *k*_2_, and *d*_2_, respectively. The heat transfer coefficient of the bottom surface of the base is *h*. The thermal resistance of the base can be expressed as the sum of one-dimensional thermal resistance and spreading thermal resistance:

(1) $$R_{\mathrm{th}2}=\frac{d_{2}}{k_{2}A_{2}}+R_{\mathrm{sp}}$$

The spreading thermal resistance can be expressed as follows:

(2) $$R_{\mathrm{sp}}=\frac{(1-\varepsilon )\phi }{\pi k_{2}r_{1}}$$

(3) $$\phi =\frac{\tanh(\lambda \tau )+\frac{\lambda }{Bi}}{1+\frac{\lambda }{Bi}\tanh(\lambda \tau )}=\tanh\left(\lambda \tau +\mathrm{arc}\tanh\left(\frac{\lambda }{Bi}\right)\right)$$

(4) $$\lambda =\pi +\frac{1}{\varepsilon \sqrt{\pi }}\;\;\;\;\;\;Bi=\frac{hr_{2}}{k_{2}}$$

(5) $$\tau =\frac{d_{2}}{r_{2}}\;\;\;\;\;\;\varepsilon =\frac{r_{1}}{r_{2}}$$

(6) $$\left\{\begin{array}{l} r_{1}=\sqrt{\frac{A_{1}}{\pi }}\\ r_{2}=\sqrt{\frac{A_{2}}{\pi }} \end{array}\right.$$

**Figure 4:**
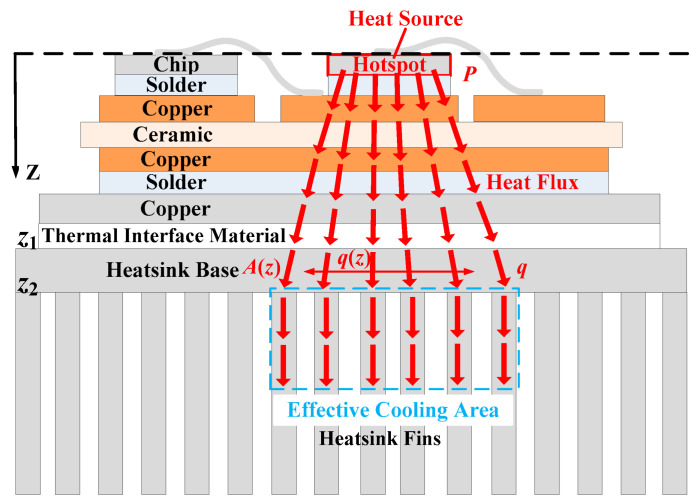
Thermal path of a traditional heatsink.

The thermal path of a traditional heatsink is shown in Figure 4. The component of the heat flux in the Z direction can be expressed as follows:

(7) $$q_{z}(z)=\frac{P}{A(z)}$$

where *P* is the power loss of the hotspot chip, and *A*(*z*) is the effective thermal conduction area in the heatsink base. The temperature increase in the heatsink base can be expressed as follows:

(8) $$\Delta T=P\cdot R_{\mathrm{th}2}=\int_{z_{1}}^{z_{2}}P\cdot \frac{dz}{k_{2}A(z)}=\int_{z_{1}}^{z_{2}}\frac{q_{z}(z)dz}{k_{2}}$$

where *z*_1_ and *z*_2_ are the Z coordinates of the upper and lower surfaces of the heatsink’s base [19].

The spreading thermal resistance reflects the resistance of the heatsink base to lateral heat transfer. According to (2), the low thermal conductivity of aluminum (*k*_2_ about 200 W/K·m) leads to greater spreading thermal resistance, resulting in weaker lateral heat transfer effects and a less effective thermal conduction area. Hence, the heat flux in the heatsink base is higher. It can be seen from (8) that the temperature increase in the traditional heatsink base is greater.

Moreover, as shown in Figure 4, heat cannot reach the lower part of the fins because of the low thermal conductivity of aluminum. Hence, the effective cooling area of the traditional heatsink fins is also smaller.

Hence, a traditional air-cooling heatsink requires optimization.

### 2.2. Principle and Classification of Heat Pipes

A heat pipe consists of an evaporation section, an adiabatic section, and a condensing section. The heat pipe is filled with water and evacuated. Water can absorb heat and boil in the evaporation section at 30 °C. Then, vapor releases its latent heat and condenses when it comes into contact with the cooler condensing section. After that, the condensed working fluid flows back to the evaporation section for the next cycle. 

According to the difference in the driving forces of the condensed working fluid flowing back to the evaporation section, heat pipes can be divided into gravity heat pipes and capillary heat pipes.

A gravity heat pipe is composed of a copper shell and a vapor core without a wick structure. The position of the evaporation section must be lower than that of the condensing section when the gravity heat pipe is in operation. The condensed liquid relies on gravity to return to the evaporation section [20].

**Figure 5:**
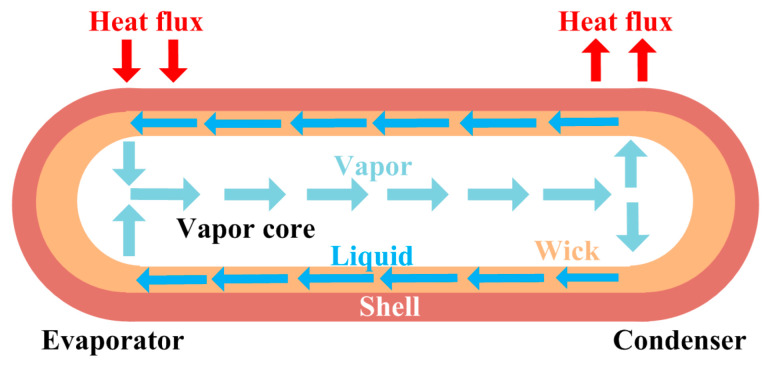
Structure and principle of capillary heat pipes.

Capillary heat pipes are needed in situations where there is no gravity or gravity needs to be overcome. The structure and principle of capillary heat pipes are shown in Figure 5. The wick structure is added between the copper shell and the vapor core. The condensed liquid returns to the evaporation section under capillary forces provided by the wick. According to the wick structure type, capillary heat pipes can be divided into grooved heat pipes, meshed heat pipes, and sintered heat pipes [21].

**Figure 6:**
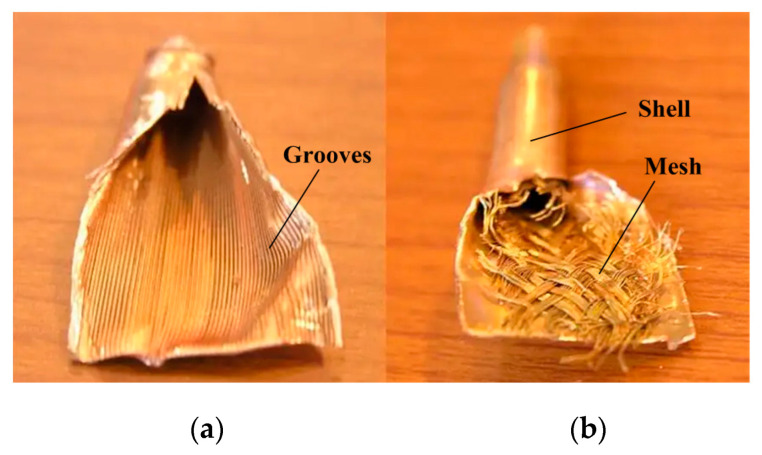
Structure of a (**a**) grooved heat pipe and (**b**) meshed heat pipe.

The structure of a grooved heat pipe is shown in Figure 6a. The wick of a grooved heat pipe has some grooves with pre-planned widths and depths. A grooved heat pipe is directional, and the direction of capillary forces is strictly limited. The performance of the grooved heat pipe is easily affected by gravity and bending.

The structure of meshed heat pipes is shown in Figure 6b. The wick of a meshed heat pipe comprises the mesh positioned close to the inside of the shell. However, mesh heat pipes have the disadvantages of high flow-back resistance and high radial thermal resistance. Moreover, meshed heat pipes are still not resistant to bending.

**Figure 7:**
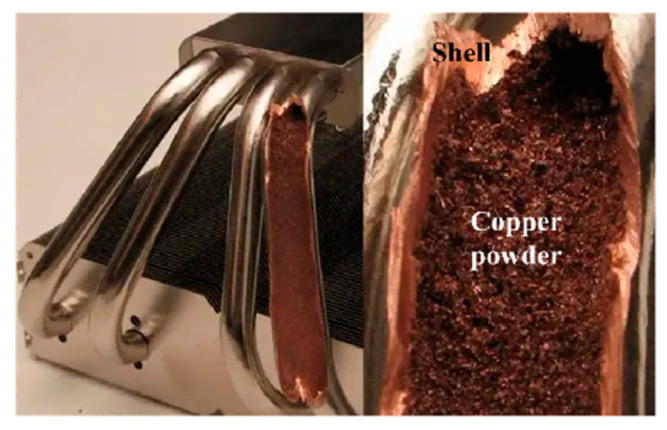
Structure of a sintered heat pipe.

The structure of sintered heat pipes is shown in Figure 7. The wick of a sintered heat pipe is the copper powder close to the inside of the shell. The performance of the sintered heat pipe is not affected by gravity and bending. 

Hence, sintered heat pipes are used to optimize the air-cooling heatsink used in this paper.

### 2.3. Research on Parameter Optimization of Straight Heat Pipes

**Figure 8:**
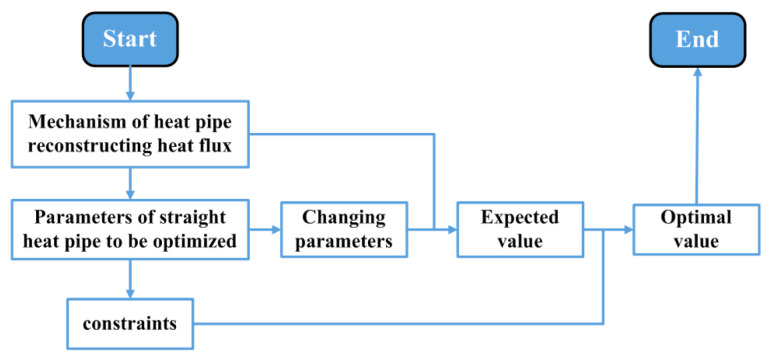
Procedure of the research on parameter optimization.

Embedding straight heat pipes in the heatsink base is a common scheme. However, as shown in Figure 2b, the existing IGBT module’s heatsink base is filled with as many heat pipes as possible, and it lacks a scientific optimization process. Hence, a parameter optimization method of the embedded straight heat pipes is researched in this section. The research procedure is shown in Figure 8. The research work is divided into the following steps. 

**Figure 9:**
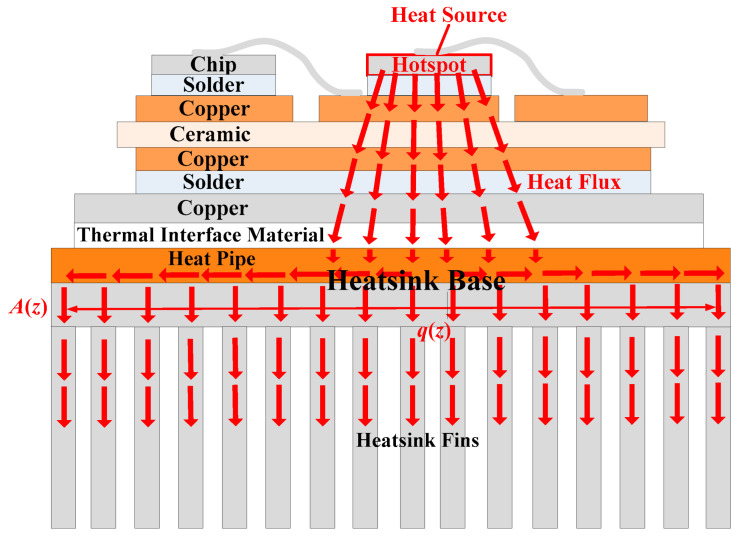
Thermal path of a heatsink with embedded straight heat pipes.

Firstly, the mechanism of heat flux reconstruction using a heat pipe is analyzed. The thermal path of a heatsink with embedded straight heat pipes is shown in Figure 9. The thermal resistance of the base can be expressed as

(9) $$R_{\mathrm{th}2}=\frac{d_{2}}{k_{R}A_{2}}+\frac{(1-\varepsilon )\phi }{\pi k_{A}r_{1}}$$

where *k*_R_ and *k*_A_ are the radial and axial thermal conductivity of the heat pipes, respectively. Since the heat pipe has extremely high axial thermal conductivity (*k*_A_ about 20,000~30,000 W/K·m), the spreading thermal resistance of the base is significantly reduced. The strong transverse heat transfer effect significantly increases the effective thermal conduction area of the heatsink’s base and reduces the heat flux. The heat generated by the chip is transferred from the top to the bottom. Heat pipes can spread heat throughout the entire base. Hence, the temperature increase in the heatsink base can be reduced.

Secondly, the types of physical parameters of straight heat pipes to be optimized need to be determined. The heat flux reconstruction mechanism is as follows: Heat pipes receive heat and transfer it to a distant location. Then, the behavior of heat pipes in reconstructing heat fluxes can be divided into heat reception behavior and heat transfer behavior based on a spatiotemporal sequence. Thus, the heat pipe parameters that affect heat reception and heat transfer behaviors are the parameters to be optimized. Evidently, the heat pipe’s diameter, the horizontal relative position between the heat pipe and the chip, and the number of heat pipes have an impact on heat reception behavior. Meanwhile, the heat pipe’s length and direction affect heat transfer behavior.

Hence, the parameters to be optimized are the position, diameter, quantity, length, and direction of the straight heat pipes.

**Figure 10:**
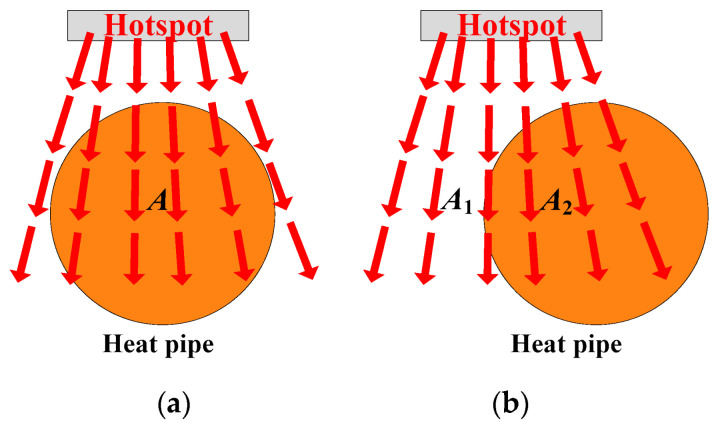
Analysis of the heat pipe position. (**a**) No deviation and (**b**) deviation.

Thirdly, the effect of the changes in these parameters on the reconstruction of heat flux is analyzed. Thus, the expected values of the parameters can be obtained:

(1) Position: Position here refers to the relative horizontal position between the axis of the heat pipe and the axis of the hotspot chip. As shown in Figure 10, when the horizontal distance between the heat pipe’s axis and the hotspot chip axis is 0 (the heat pipe is arranged directly under the hotspot chip), the heat pipe can cover the thermal path from the hotspot chip to the heatsink base as extensively as possible. Hence, the heat pipe can absorb and spread the heat of the hotspot chip, reducing the heat flux as much as possible. As the two axes deviate, the heat pipe cannot fully cover the thermal path. Therefore, the effectiveness of heat pipes in absorbing and transmitting heat gradually weakens.

After qualitative analysis, quantitative analyses are conducted. As the thermal path is covered by the heat pipe entirely, the axial thermal resistance of the heatsink based on the thermal path of Figure 10a can be expressed as

(10) $$R_{\text{th-ax}(a)}=\frac{d}{k_{\mathrm{ax}}A}$$

where *k*_ax_ is the axial thermal conductivity of the heat pipe, *A* is the cross-sectional area of the thermal path in the base, and *d* is the horizontal distance from the hotspot chip to one end of the heat pipe [22].

As the two axes deviate, in the horizontal direction, the thermal resistance of the heat pipe is parallel to the thermal resistance of the aluminum base. The axial thermal resistance of the heatsink based on the thermal path of Figure 10b can be expressed as

(11) $$\left\{\begin{array}{l} R_{\text{th-ax}(b)}=\frac{d}{k_{\mathrm{eq}}A}\\ \frac{1}{R_{\text{th-ax}(b)}}=\frac{k_{\mathrm{Al}}A_{1}}{d}+\frac{k_{\mathrm{ax}}A_{2}}{d}\\ A=A_{1}+A_{2}\\ k_{\mathrm{eq}}=k_{\mathrm{Al}}+(k_{\mathrm{ax}}-k_{\mathrm{Al}})\frac{A_{2}}{A} \end{array}\right.$$

where *k*_eq_ is the equivalent axial thermal conductivity of the heatsink based on the thermal path of Figure 10b, *k*_Al_ is the thermal conductivity of aluminum, and *A*_1_ and *A*_2_ are the cross-sectional area of the aluminum base and heat pipe on the thermal path. As the two axes deviate, *A*_2_ becomes smaller, and *k*_eq_ is reduced.

Hence, the horizontal distance between the heat pipe axis and the hotspot chip axis is supposed to be 0.

**Figure 11:**
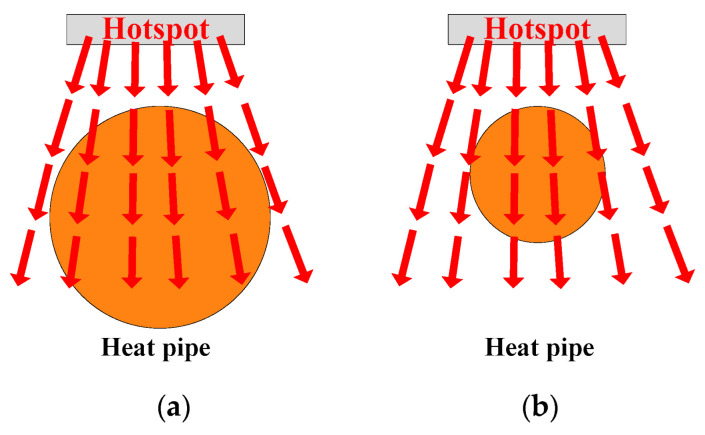
Analysis of the heat pipe diameter. (**a**) Thicker and (**b**) thinner.

(2) Diameter: As shown in Figure 11, a thicker heat pipe can cover the thermal path as extensively as possible. Similarly, the heat pipe can absorb and spread the heat of the hotspot chip, reducing the heat flux as much as possible. As the heat pipe gradually becomes thinner, the heat pipe cannot fully cover the thermal path. Thus, the effectiveness of heat pipes in absorbing and transmitting heat gradually weakens.

**Figure 12:**
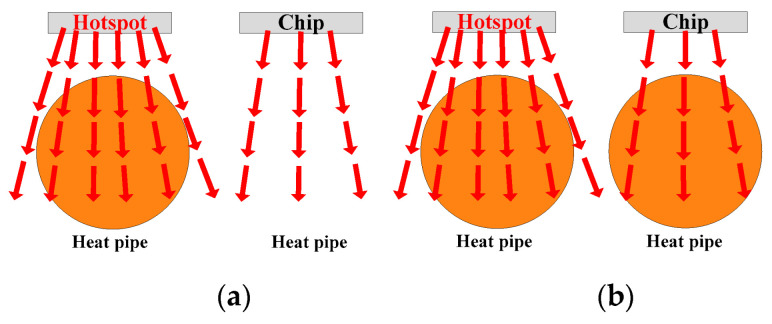
Analysis of the heat pipe quantity. (**a**) Same as the number of hotspot chips and (**b**) as many heat pipes as possible.

The quantitative analysis of diameter is the same as that for position. Hence, the heat pipes are expected to be as thick as possible:

(3) Quantity: As can be seen in Figure 12, a heat pipe that is directly under the hotspot chip and with maximum thickness can fully cover the thermal path. If some heat pipes are added, additional heat pipes will be located below normal chips with lower power losses. As additional heat pipes are not on the thermal path of the hotspot chip, they have little effect on reducing the heat flux of hotspot chips.

Hence, the quantity of the heat pipes is expected to be the same as that of the hotspot chips.

**Figure 13:**
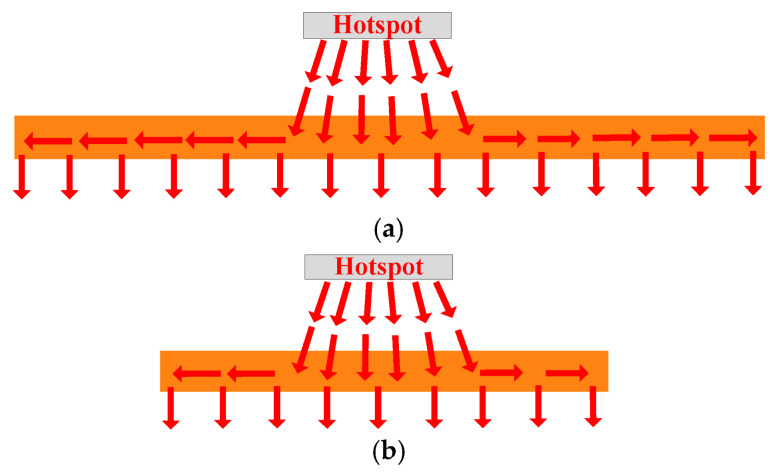
Analysis of the heat pipe length. (**a**) Longer and (**b**) shorter.

(4) Length: As shown in Figure 13, a longer heat pipe can transfer heat further away, resulting in a larger effective thermal conduction area. And the heat flux of the heatsink base will be lower.

Hence, heat pipes should be designed to be as long as possible.

(5) Direction: Direction here refers to the positional relationship between the heat pipe and the heatsink’s fins. The first positional relationship is that the heat pipe is perpendicular to the fins. Another positional relationship is that the heat pipe is parallel to the fins. Moreover, the direction of the straight heat pipe has little effect on heat flux reconstruction.

**Figure 14:**
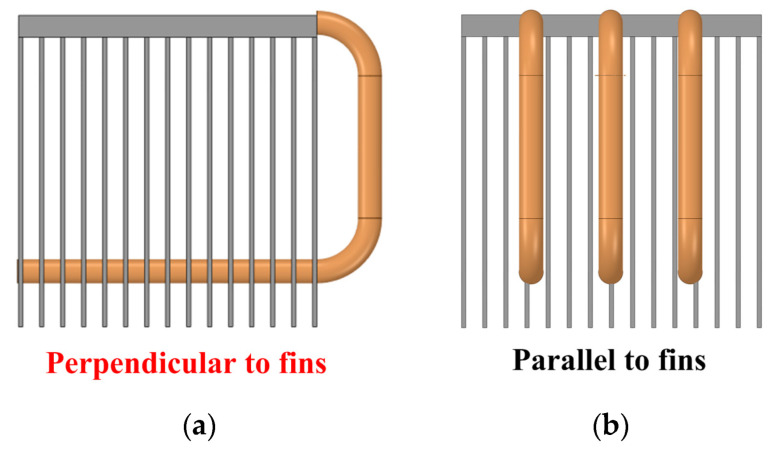
Constraints on the heat pipe direction. (**a**) Perpendicular and (**b**) parallel to the fins.

Fourth, the constraints on the values of these parameters are studied in accordance with the extensive heat pipe heatsink design experience accumulated in earlier stages:

(1) Diameter: Heat pipes with a diameter of more than 10 mm have high mechanical strength and are not easy to bend. Even if force is applied to bend them, the capillary structure at the bent part will be damaged, thereby reducing heat transfer performance and being unfavorable for subsequent special-shaped design.
(2) Length: The length of the straight heat pipe should not be greater than the side length of the heatsink.
(3) Direction: As shown in Figure 14a, the heat pipes should be perpendicular to the fins. Heat pipes parallel to the fins cannot pass through the fins, thus making subsequent special-shaped heat pipe design difficult, as shown in Figure 14b.

Finally, the optimal value of each parameter can be obtained according to the expected value and constraint. The straight heat pipes should be placed directly under the hotspot chips. The diameter of the straight heat pipes should be 10 mm. And the quantity of heat pipes should be the same as the quantity of hotspot chips. The length of the straight heat pipe should be the same as the side length of the heatsink. Finally, the straight heat pipe should be perpendicular to the fins.

### 2.4. Research on Special-Shaped Design of Heat Pipes

After optimizing the parameters of straight heat pipes, the design of special-shaped heat pipes is carried out. Special-shaped design refers to the design of the shape of the parts of the heat pipes that protrude from the heatsink base. The purpose of special-shaped designs is to connect the heat pipes to the fins, thereby transferring heat to the fins.

As shown in Figure 2a, U-shaped heat pipes are used in existing computer CPU heatsinks. This heatsink type conducts heat only using heat pipes, and the thermal path of the traditional heatsink in Figure 4 is not used. 

However, if both the thermal paths of heat pipes and traditional heatsinks are used, the thermal resistance of the heatsink can be further reduced based on the parallel thermal resistance theory. This is because thermal resistance in parallel is analogous to electrical resistance in parallel.

**Figure 15:**
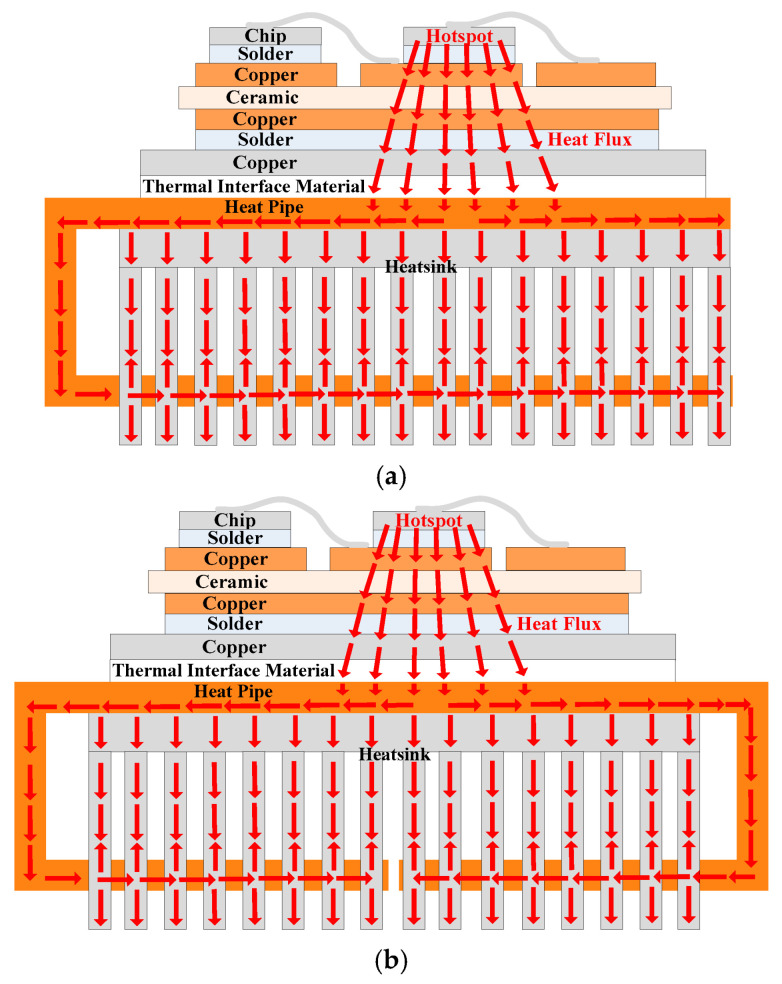
Special-shaped design of (**a**) U-shaped and (**b**) C-shaped heat pipes.

The special-shaped design is shown in Figure 15. It can be seen in Figure 15a that the traditional thermal path is retained. Moreover, U-shaped heat pipes add a new thermal path with much lower thermal resistance. Adding new thermal paths reflects the idea of enhanced cooling. The thermal resistance of the U-shaped heat pipe heatsink can be expressed as

(12) $$R_{\text{th-U}}=\frac{1}{\frac{1}{R_{\text{th-hp}}}+\frac{1}{R_{\text{th-H}}}}=\frac{R_{\text{th-hp}}}{1+\frac{R_{\text{th-hp}}}{R_{\text{th-H}}}}$$

where *R*_th-hp_ is the thermal resistance of the heat pipe, and *R*_th-H_ is the thermal resistance of the traditional heatsink. It can be seen that the total thermal resistance of the heatsink is lower than the thermal resistance of the heat pipes.

As shown in Figure 15b, the C-shaped heat pipe can be divided into two sections, and the two sections are still in parallel. Hence, the thermal resistance of the C-shaped heat pipe heatsink can be expressed as

(13) $$R_{\text{th-U}}=\frac{1}{\frac{1}{R_{\text{th-hp}}}+\frac{1}{R_{\text{th-hp}}}+\frac{1}{R_{\text{th-H}}}}=\frac{R_{\text{th-hp}}}{2+\frac{R_{\text{th-hp}}}{R_{\text{th-H}}}}$$

The thermal resistance of the C-shaped heat pipe heatsink can be further reduced compared to the U-shaped heat pipe.

Hence, the C-shaped heat pipe is selected.

### 2.5. Convenient Optimization Method for Heat Pipe Heatsinks

**Figure 16:**
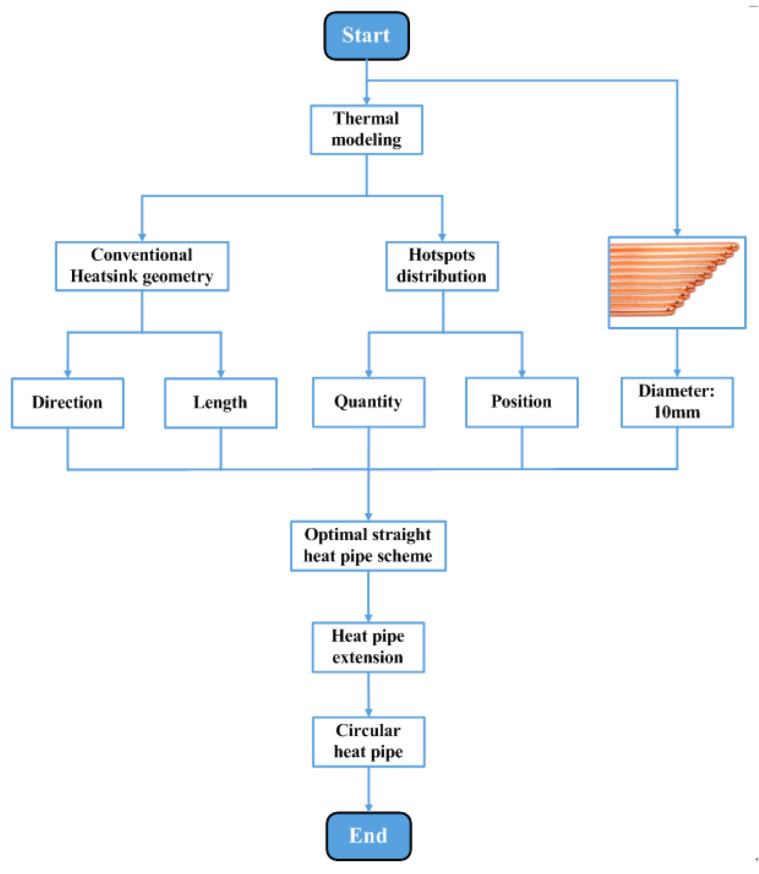
Procedure of the convenient optimization method for heat pipe heatsinks.

After the research on the parameter optimization and special-shaped design of heat pipes, a convenient optimization method for heat pipe heatsinks can be proposed. The procedure of the method is shown in Figure 16.

Firstly, the geometry of the conventional heatsink and hotspot chip distributions can be obtained through thermal modeling.

Secondly, the direction and length of the straight heat pipes can be determined according to the heatsink’s geometry. The length of the straight heat pipes should be the same as the side length of the heatsink, and the straight heat pipes should be perpendicular to the fins. The quantity and position of the straight heat pipes can be determined according to the hotspot chips’ distribution. The straight heat pipes should be placed directly under the hotspot chips, and the quantity of heat pipes should be the same as that of the hotspot chips. The diameter of the straight heat pipes should be 10 mm. Thus, the optimal scheme of straight heat pipes can be obtained.

Finally, the heat pipes are extended to form a C shape. 

## 3. Case Study

In this section, an air-cooling heatsink of the IGBT module in the MMC-based motor drive device is optimized based on the proposed method. As indicated in many references, currently, MMC is the most typical case of thermal imbalance with respect to hotspot chips inside power modules. The detailed information and reasons for thermal imbalance in hotspot chips can be found in [23,24,25,26].

### 3.1. Description of the MMC System

**Figure 17:**
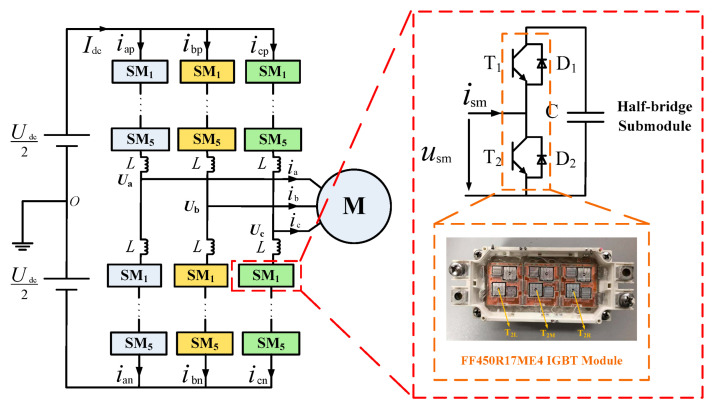
Circuit configuration of the MMC system.

A circuit configuration of the MMC system is shown in Figure 17. Each arm consists of five submodules (SMs) and an arm inductor. Each SM has a half-bridge configuration with an SM capacitor. The system’s parameters are presented in Table 1. Infineon half-bridge IGBT modules, FF450R17ME4, are used in the MMC system.

### 3.2. Situation of Using Conventional Heatsinks (Control Group)

**Figure 18:**
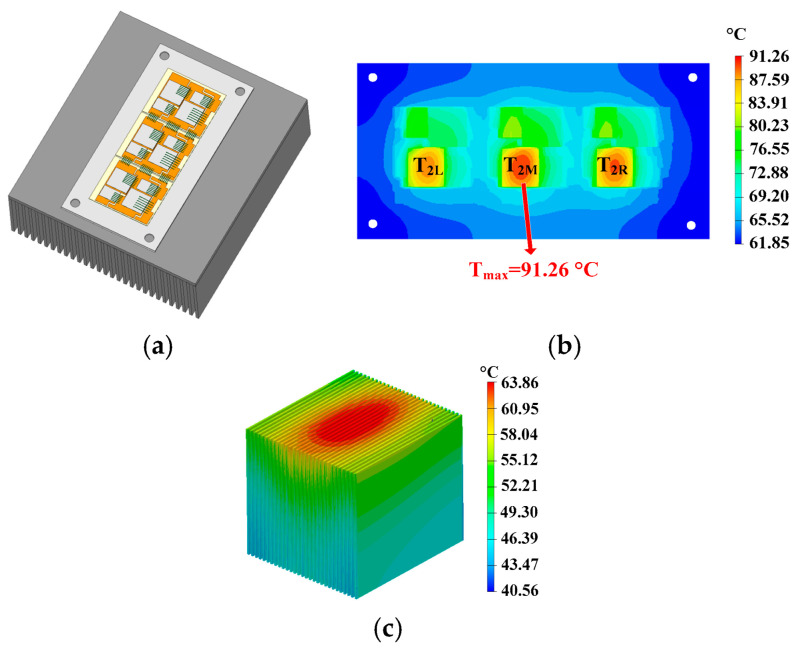
(**a**) Geometry, (**b**) hotpot distribution, and (**c**) temperature distribution of the conventional heatsink (control group).

The thermal modeling of the IGBT module with a conventional heatsink (control group) is presented in this section. The geometry is shown in Figure 18a. The upper surface of the heatsink is square-shaped.

The current of the devices can be obtained using MATLAB 2018 Simulink. The average power loss of the devices can be calculated according to the power loss models in [27]. For FF450R17ME4, each device in the half-bridge structure is composed of three chips in parallel. The chips are set as heat sources in the thermal simulation. The ambient temperature is 25 °C, and the air flow rate of the fan is 35CFM. The upper surface of the power module is thermally insulated in the simulation. The material properties of the power module are shown in Table 2, where TIM is the thermal interface material, and CTE is the coefficient of thermal expansion.

The hotspot chips’ distribution is shown in Figure 18b. There are three hotspot chips in the power module, and the temperature of the middle hotspot chip is the highest. The highest temperature is 91.26 °C. The temperature distribution of the heatsink is shown in Figure 18c. It can be seen that the temperature of the middle section of the heatsink’s base plate is directly below the power module, and it is located much higher than the other section. Furthermore, the temperature of the lower part of the fins is lower than that of the upper section. 

This indicates that due to the low thermal conductivity of the conventional heatsink, heat accumulates in the middle section of the heatsink and cannot reach the lower section of the fins. This results in a lower utilization rate of the heatsink. The thermal simulation result of the conventional heatsink, as shown in Figure 18c, is consistent with the theoretical analysis results in Part A of Section 2 (as shown in Figure 4).

### 3.3. Parameters Optimization of Straight Heat Pipes

The parameters of straight heat pipes are optimized based on 3D modeling and thermal simulation in this section.

(1) Position

**Figure 19:**
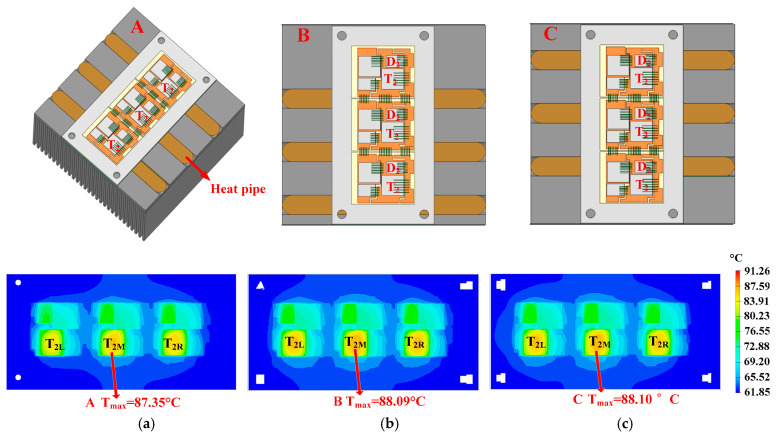
Optimization of positional parameters: (**a**) 87.35 °C, (**b**) 88.09 °C, and (**c**) 88.10 °C.

The optimization of positional parameters is shown in Figure 19. In heatsink A, the horizontal distance between the heat pipe axis and the hotspot chip (T_2_) axis is 0 (the heat pipe is arranged directly under the hotspot chip). In heatsinks B and C, the horizontal distance between the heat pipe axis and the hotspot chip (T_2_) axis is 12 mm. According to the principle of controlling variables, other parameters of the heat pipe are the same.

**Figure 20:**
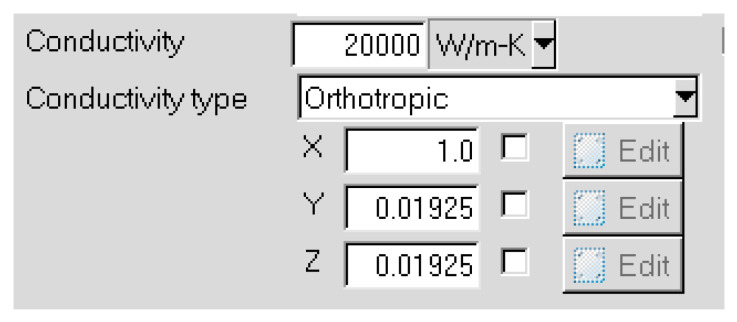
Settings of the thermal conductivity of heat pipes in finite element thermal simulation software.

The setting of the thermal conductivity of heat pipes in finite element thermal simulation software is shown in Figure 20. The conductivity type of heat pipes is orthotropic. This means that heat pipes have different thermal conductivity coefficients in various directions. According to [28,29,30,31], the axial (X direction) thermal conductivity of heat pipes is 20,000 W/K·m. The radial (Y and Z direction) thermal conductivity is the same as copper (385 W/K·m).

As shown in Figure 19, the hotspot chip temperatures of the power module with heatsinks A, B, and C are 87.35 °C, 88.09 °C, and 88.10 °C, respectively. Compared with the situation of using a conventional heatsink, straight heat pipes can reduce the hotspot chip’s temperature. Most importantly, placing the heat pipe directly below the hotspot chip has the best cooling effect.

(2) Diameter

**Figure 21:**
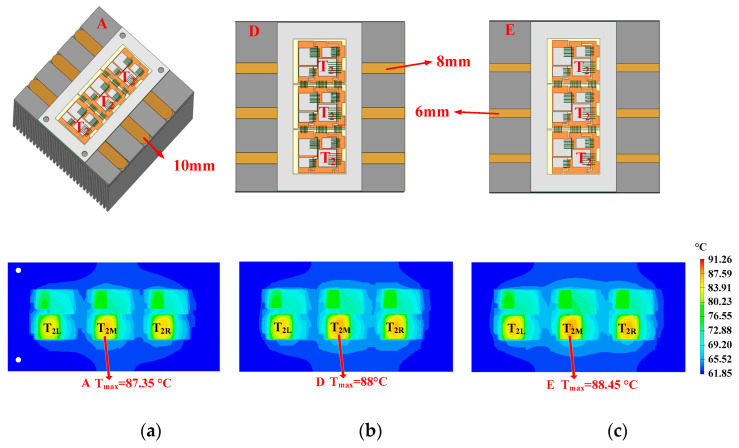
Optimization of diameter: (**a**) 10, (**b**) 8, and (**c**) 6 mm.

The optimization of the diameter is shown in Figure 21. In heatsinks A, D, and E, the diameters of the heat pipes are 10 mm, 8 mm, and 6 mm, respectively. The hotspot chip temperatures of the power module with heatsinks A, D, and E are 87.35 °C, 88 °C, and 88.45 °C, respectively. Hence, using a thicker heat pipe has a better cooling effect. Considering the constraint that heat pipes with a diameter greater than 10 mm are difficult to bend, heat pipes with a diameter of 10 mm are selected.

(3) Quantity

**Figure 22:**
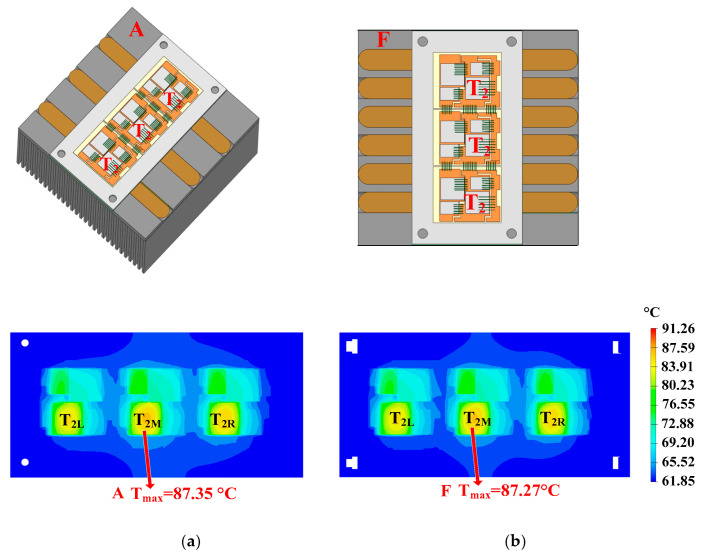
Optimization of quantity: (**a**) three and (**b**) six heat pipes.

The optimization of quantity is shown in Figure 22. In heatsink A, there are three heat pipes. The quantity of the heat pipes is the same as that of the hotspot chips. In heatsink F, there are six heat pipes. Heatsink F is filled with as many heat pipes as possible. The hotspot chip temperatures of the power module with heatsinks A and F are 87.35 °C and 87.27 °C, respectively. It can be seen that the difference in hotspot chip temperatures between the two situations is very small. Hence, considering cost–performance, the quantity of the heat pipes should be the same as that of the hotspot chips.

(4) Length

**Figure 23:**
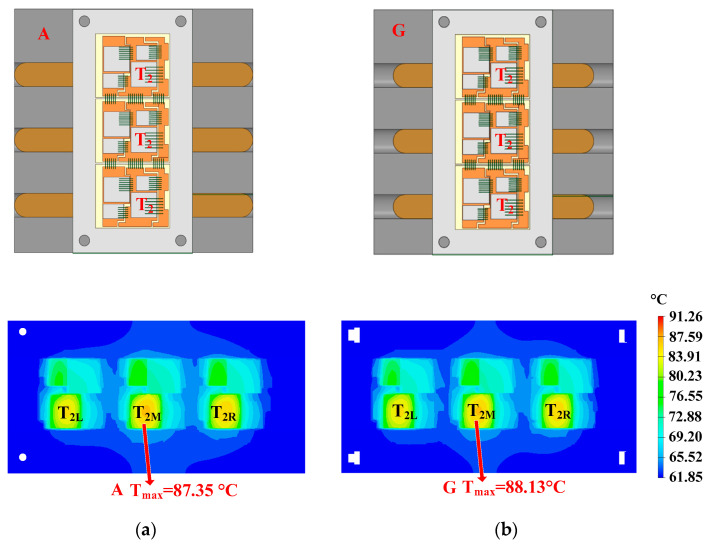
Optimization of length: (**a**) 122 mm and (**b**) 102 mm.

The optimization of length is shown in Figure 23. In heatsink A, the length of the heat pipes is 122 mm, while the length of the heat pipes is 102 mm in heatsink G. The hotspot chip temperatures of the power module with heatsinks A and G are 87.35 °C and 88.13 °C, respectively. It can be seen that using longer heat pipes has a better cooling effect. Considering the constraint of the heatsink’s size, the length of the heat pipe should be the same as the side length of the heatsink.

(5) Direction

**Figure 24:**
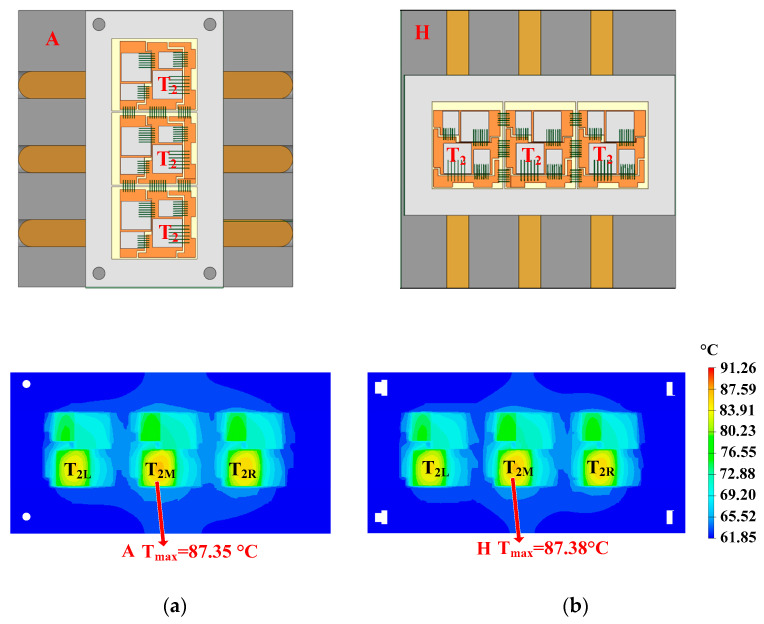
Optimization of direction. (**a**) Perpendicular and (**b**) parallel to the fins.

The optimization of direction is shown in Figure 24. In heatsink A, the heat pipes are perpendicular to the fins, while the heat pipes are parallel to the fins in heatsink H. The heatsink here has a square structure, which means that changing the direction of the heat pipes does not change their length. This meets the requirement of controlling variables. The hotspot chip temperatures of the power module with heatsinks A and H are 87.35 °C and 87.38 °C, respectively. It can be seen that the direction of the straight heat pipe has little effect on the hotspot chip’s temperature. Considering the constraint that heat pipes need to pass through the fins in the subsequent special-shape design stage, they should be perpendicular to the fins.

In summary, the finite element simulation results are consistent with the theoretical analysis results in Part C of Section 2. Heatsink A is the optimal solution for the parameters optimization part of this case study. The parameters of the heat pipes in heatsink A are shown in Table 3.

### 3.4. Special-Shaped Design of Heat Pipes

**Figure 25:**
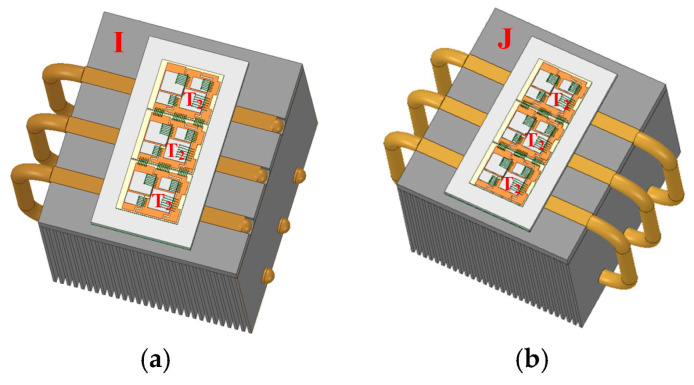
Optimized heatsinks using (**a**) U-shaped and (**b**) C-shaped heat pipes.

**Figure 26:**
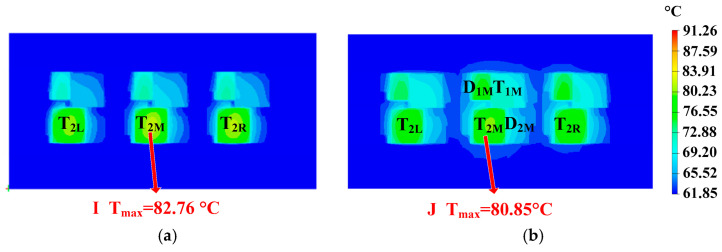
Temperature distribution of the power modules using (**a**) heatsinks I and (**b**) J.

In this section, the design of special-shaped heat pipes is carried out based on heatsink A. Heatsinks I and H are shown in Figure 25. Three U-shaped heat pipes are used in heatsink I, while three C-shaped heat pipes are used in heatsink J. The heat pipes pass through the lower part of the fins. The temperature distributions of the power modules using heatsinks I and J are shown in Figure 26. The hotspot chip temperatures of the power module with heatsinks I and J are 82.76 °C and 80.85 °C, respectively. It can be seen that special-shaped heat pipes can further reduce the hotspot chip’s temperature. Moreover, compared with U-shaped heat pipes, C-shaped heat pipes have better cooling effects, which is consistent with the theoretical analysis results in Part D of Section 2.

## 4. Experiment

**Figure 27:**
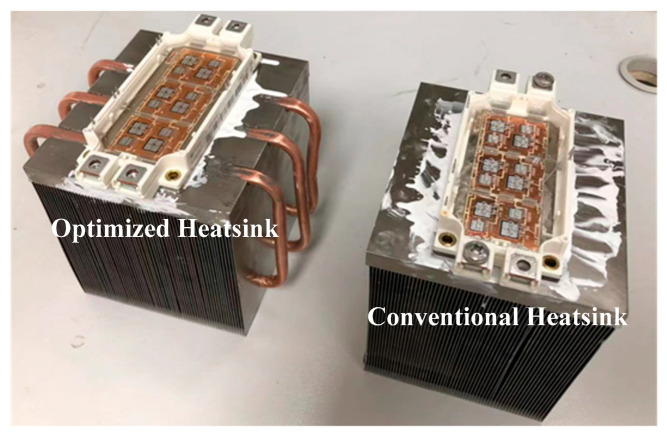
Physical photographs of the optimized heatsink and conventional heatsink.

In this section, in order to conduct experimental verification, heatsink J and a conventional heatsink are processed, as shown in Figure 27. The conventional heatsink is identical to the optimized heatsink in all other aspects except for the absence of heat pipes. The effectiveness of the proposed enhanced cooling method will be verified by comparing the hotspot chip temperatures of the power module using these two types of heatsinks.

Since an infrared thermal imager is used for temperature measurements, it is necessary to remove the silicone gel from the power module to ensure the accuracy of temperature measurements. Consequently, the insulation level of the module no longer meets the operational requirements of the MMC prototype. Therefore, an equivalent thermal test bench is adopted to simulate the steady-state thermal distribution of the IGBT module under MMC operating conditions. Since this is a steady-state experiment, it is sufficient to ensure that the average value of the device’s conduction loss in the experiment is equal to the average value of the actual device’s loss under MMC operating conditions.

**Figure 28:**
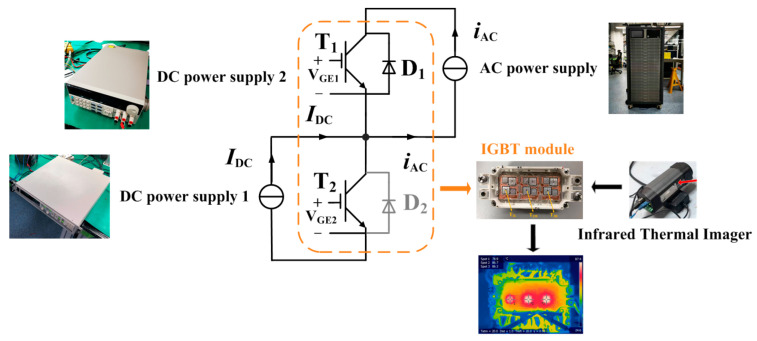
Schematic diagram of the experimental platform.

The schematic diagram of the experimental circuit is shown in Figure 28. As the average currents of T1 and D1 are the same and their power losses are similar in MMC, they are heated by the sinusoidal current output from a high-power AC power supply (ACTION A116): The current flows through T1 during the positive half-cycle and through D1 during the negative half-cycle. The driving voltage of T1 is set to 20 V. As indicated in the IGBT module datasheet, when the driving voltage is 20 V, the static characteristics of the IGBT are essentially consistent with those of the anti-parallel diode. When a sinusoidal current passes through them, their conduction losses are basically the same. The rms (root mean square) value of the AC power supply’s output current is adjusted to 68 A, ensuring that the loss values of T1 and D1 are roughly equal to those in the thermal simulation. 

T2 is heated by DC power supply 1 (model: IT6525D; specifications: 500 V/20 A/3 kW). For safety reasons, it is necessary to reduce the current in the circuit (while still maintaining high device loss). Thus, we set the driving voltage of T2 to 6.6 V to make it operate in the active region—this allows T2 to achieve a relatively large on-state voltage drop. DC power supply 1 works in a constant power mode and stably outputs 280 W of power. Under actual MMC operating conditions, compared with other devices, the loss of D2 is very small and is negligible. Therefore, to simplify the experimental platform, D2 is not connected to the circuit. This approximation has a negligible impact on the experimental results.

The driving voltages of T1 and T2 are both provided by DC power supply 2 (model: IT6302).

**Figure 29:**
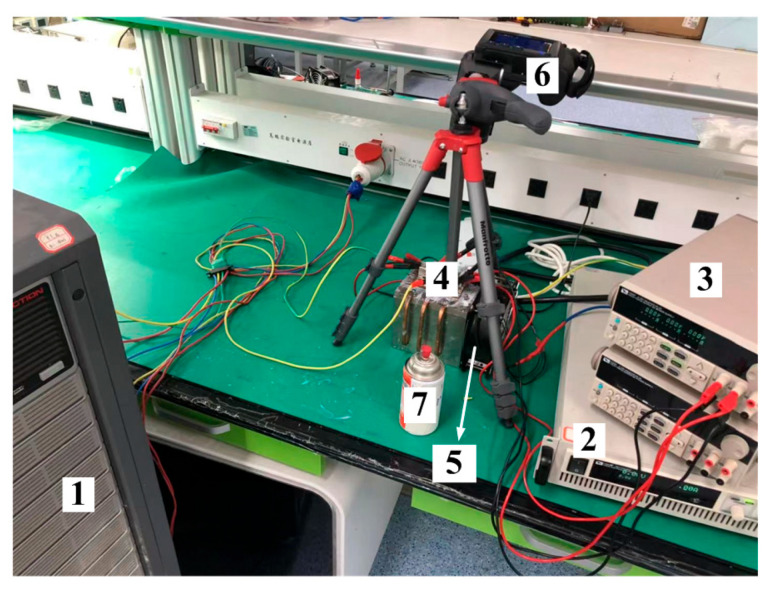
Experimental platform.

The experimental platform is shown in Figure 29. From 1 to 7, the following are shown: AC power supply; DC power supply 1; DC power supply 2; heatsink J with C-shaped heat pipes; fan; infrared thermal imager; and special black pigment, respectively. In the experiment, a model ADDA AA1282HB-AW fan (air flow rate: 35CFM) was used to cool the IGBT module. The fan has dimensions of 120 mm × 120 mm, which match the dimensions of the windward cross-section of the heatsink. The silicone gel of the power module is removed by a special solvent. Then, the power module is sprayed with a special black pigment. Finally, the temperature of the power module is measured by an FLIR infrared thermal imager T60SC (±0.1 °C accuracy). The room temperature of the laboratory is 25 °C.

**Figure 30:**
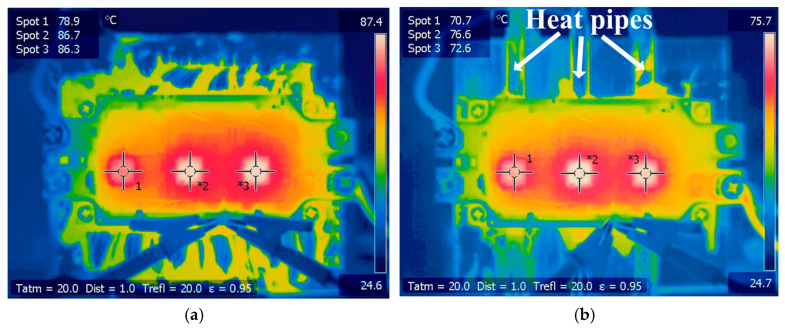
Thermal images of the IGBT module using (**a**) a conventional heatsink and (**b**) heatsink J.

The thermal images of the IGBT module using a conventional heatsink and heatsink J are shown in Figure 30. The hotspot chip temperatures of the IGBT module using conventional heatsinks and heatsink J are 86.7 °C and 76.6 °C, respectively. It can be seen that after adopting the enhanced cooling method, the hotspot chip temperature can be reduced by 10.1 °C, a decrease of 11.65%. Therefore, the reliability of the power module will be significantly improved.

Furthermore, the hotspot chip temperatures in the experiment are slightly lower than those in the simulation. The reason is as follows: In the thermal simulation, to replicate the actual application scenario, we set the upper surface of the power module to an adiabatic state. This is because, under practical operating conditions, the upper surface of the power module is filled with silicone gel—the purpose of filling the module with silicone gel is to meet insulation requirements, and silicone gel has adiabatic properties. Therefore, the heat generated by the chip can only be transferred downward to the copper substrate and heat sink, and it cannot dissipate from the top of the chip. However, in the experiment, the silicone gel was removed, and the upper surface of the power module was no longer in an adiabatic state, leading to partial heat dissipation from the top of the chip. This is exactly why the chip temperature in the experiment is lower than that in the thermal simulation results.

## 5. Conclusions

In this paper, an enhanced cooling method for multi-chip power modules with uneven power loss distribution on all-electric propulsion ships based on parameter optimization and the special-shaped design of sintered heat pipes is proposed. This paper draws the following conclusions:

(1) The position, diameter, quantity, length, and direction of the heat pipes are the parameters to be optimized.
(2) A. The horizontal distance between the heat pipe axis and the hotspot chip axis is supposed to be 0.
  B. The heat pipes are expected to have the maximum possible thickness.
  C. The quantity of the heat pipes is expected to be the same as that of the hotspot chips.
  D. The heat pipes should have the maximum possible length.
(3) There are also some constraints with respect to diameter, length, and direction. The heat pipe’s diameter should not be greater than 10 mm. The length of the straight heat pipe should not be greater than the side length of the heatsink. The heat pipes should be perpendicular to the fins.
(4) Designing special-shaped heat pipes can further reduce the thermal resistance of heatsinks by adding parallel thermal resistance to the existing thermal path. Among them, the C-shaped heat pipe has the best cooling effect.

The above conclusions can constitute a convenient optimization method for a heat pipe heatsink applicable to various power electronic converters. 

The proposed enhanced cooling method was verified by a case study based on an MMC inverter. In the simulation, the hotspot chip’s temperature decreased from 91.26 °C to 80.85 °C, while the hotspot chip temperature decreased from 86.7 °C to 76.6 °C in the experiment. The agreement between the experiment and simulation is good.

## Figures and Tables

**Table 1:** Parameters of the MMC System.

Symbol	Meaning	Value
*P*	System-rated active power	1.3 MW
*U*_dc_	DC-link voltage	4.5 kV
*U*_m_	Amplitude of ac-side phase voltage	2 kV
*N*	Number of SMs	5
*L*	Arm inductor	5 mH
cos*φ*	Power factor	0.9
*C*	SM capacitor	4.7 mF
*f*_s_	Switching frequency	1 k Hz
*f*	Fundamental frequency	50 Hz

**Table 2:** Material properties.

Component	Material	Thermal Conductivity (W/K·m)	Thickness (mm)	CTE (10^−6^/K)
Chip	Silicon	130	0.2	3
Chip solder	SAC305	66	0.12	23
Upper cooper	Copper	385	0.3	17
Ceramic	Alumina	25	0.38	7
Bottom copper	Copper	385	0.3	17
DBC solder	SAC305	66	0.12	23
Baseplate	Copper	385	3	17
TIM	Silicone grease	5	0.1	/
Heatsink base	Aluminum alloy	201	12 mm	23
Heatsink fins	Aluminum alloy	226	1.75 mm	24

**Table 3:** Parameters of the heat pipes in heatsink A.

Parameters	Value (Explanation)
Position	Directly under the hotspot chip
Diameter	10 mm
Quantity	3 (same as the number of the hotspot chips)
Length	122 mm (same as the side length of the heatsink)
Direction	Perpendicular to the fins

## Data Availability

The data that support the findings of this study are available from the corresponding author upon reasonable request.
